# Production Process Optimization of Recombinant *Erwinia carotovora*l-Asparaginase II in *Escherichia coli* Fed-Batch Cultures and Analysis of Antileukemic Potential

**DOI:** 10.1021/acsomega.4c04711

**Published:** 2024-08-03

**Authors:** Bruna Coelho de Andrade, Gaby Renard, Adriano Gennari, Leonardo Luís Artico, José Ricardo Teixeira Júnior, Daniel Kuhn, Priscila Pini Zenatti Salles, Claucia Fernada Volken de Souza, Gustavo Roth, Jocelei Maria Chies, José Andrés Yunes, Luiz Augusto Basso

**Affiliations:** † National Institute of Science and Technology in Tuberculosis, Research Center for Molecular and Functional Biology, Pontifical Catholic University of Rio Grande do Sul, Porto Alegre, Rio Grande do Sul 90619-900, Brazil; ‡ Graduate Program in Medicine and Health Sciences, Pontifical Catholic University of Rio Grande do Sul, Porto Alegre, Rio Grande do Sul 90619-900, Brazil; § Quatro G Pesquisa & Desenvolvimento Ltd., Porto Alegre, Rio Grande do Sul 90619-900, Brazil; ∥ Food Biotechnology Laboratory, Biotechnology Graduate Program, University of Vale do Taquari (UNIVATES), Lajeado, Rio Grande do Sul 95914-014, Brazil; ⊥ Centro Infantil Boldrini, Campinas, São Paulo 13083-210, Brazil; # Graduate Program in Genetics and Molecular Biology, Biology Institute, State University of Campinas, Campinas, São Paulo 13083-970, Brazil; ∇ Pontifical Catholic University of Rio Grande do Sul, Porto Alegre, Rio Grande do Sul 90619-900, Brazil; ¶ Department of Medical Genetics, Faculty of Medical Sciences, State University of Campinas, Campinas, São Paulo 13083-970, Brazil; ■ Graduate Program in Cellular and Molecular Biology, Pontifical Catholic University of Rio Grande do Sul, Porto Alegre, Rio Grande do Sul 90619-900, Brazil

## Abstract

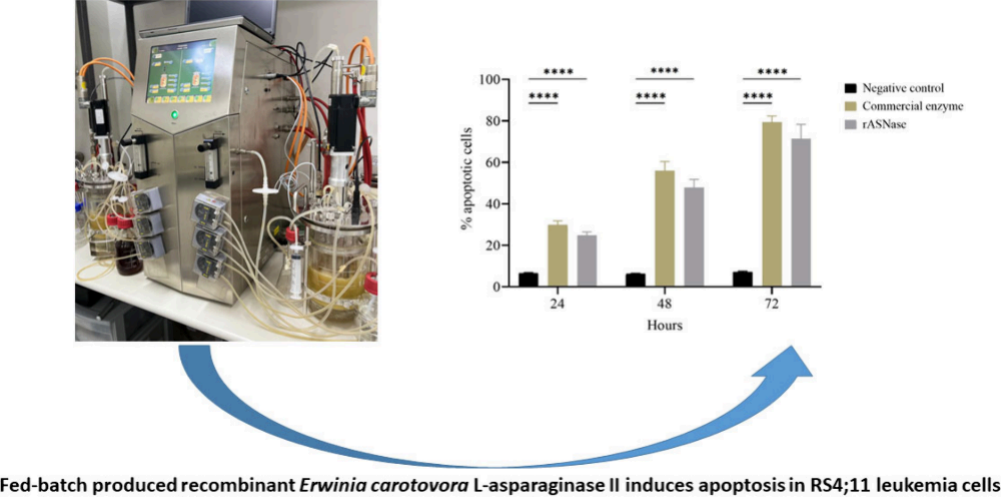

The aims of this work were to optimize the production of *Erwinia carotovora*l-asparaginase II enzyme in *Escherichia coli* by different fed-batch cultivation strategies using a benchtop bioreactor and to evaluate the therapeutic potential of the recombinant enzyme against different acute lymphoblastic leukemia cell lines. The highest enzyme activities (∼98,000 U/L) were obtained in cultures using the DO-stat feeding strategy with induction in 18 h of culture. Under these experimental conditions, the maximum values for recombinant l-asparaginase II (rASNase) yield per substrate, rASNase yield per biomass, and productivity were approximately 1204 U/g_glucose_, 3660 U/g_cells_, and 3260 U/(L·h), respectively. This condition was efficient for achieving high yields of the recombinant enzyme, which was purified and used in *in vitro* antileukemic potential tests. Of all the leukemic cell lines tested, RS4;11 showed the highest sensitivity to rASNase, with an IC_50_ value of approximately 0.0006 U/mL and more than 70% apoptotic cells. The study demonstrated that the cultivation strategies used were efficient for obtaining high yield and productivity of rASNase with therapeutic potential inasmuch as cytotoxic activity and induction of apoptosis were demonstrated for this protein.

## 1 Introduction

The l-asparaginase enzyme (l-asparagine amidohydrolase, EC 3.5.1.1) constitutes one of the drugs for the combined chemotherapy treatment of acute lymphoblastic leukemia (ALL) in children and adults.^1^ The mechanism of action of l-asparaginase in the treatment of ALL is through its hydrolysis reaction, in which the enzyme catalyzes the conversion of l-asparagine into ammonia and aspartic acid by a deamination reaction.^2,3^ The amino acid l-asparagine is not essential in normal human cells, since cells are able to synthesize asparagine from other amino acids by the action of transaminases and asparagine synthetase.^4,5^ Unlike normal cells, leukemia cells have low or no asparagine synthetase activity, thus being unable to synthesize asparagine from other amino acids and consequently making asparagine an essential amino acid for leukemia cells.^4,6^ Owing to this dependence on extracellular asparagine, when the enzyme l-asparaginase is administered and causes depletion of this amino acid, the synthesis of DNA and proteins in tumor cells is inhibited, affecting cellular functions and leading to the death of cancer cells.^7−10^

Despite being identified in various sources, only asparaginases from *Escherichia coli* and *Erwinia chrysanthemi* have been approved for use in chemotherapy.^4,8,11^*E. coli*-derived preparations have been approved by the FDA for use in ALL and are indicated in most first-line therapies; and *Erwinia* derivatives are approved for patients who develop hypersensitivity reactions to *E. coli*-derived enzymes.^7,12^ These preparations can become immunogenic, and their clinical efficacy is limited by the development of antiasparaginase antibodies which are associated with a decrease in enzyme activity and accompanied by the presence of hypersensitivity reactions.^8,13^l-Asparaginase is associated with adverse reactions, with reaction rates of 30 to 70% when the *E. coli*-derived enzyme is administered.^12,14^ Once hypersensitivity reactions occur, it is necessary to change the l-asparaginase preparation to one which is not cross-reactive. The therapy can be continued with alternative l-asparaginases until a new limiting immune reaction occurs. Enzymes derived from *Erwinia* subspecies are immunologically distinct from preparations derived from *E. coli* and consequently have different antigenic sites.^15−17^ Regarding its pharmacokinetics, *Erwinia*l-asparaginase has a shorter half-life compared to other preparations, requiring it to be administered in larger and more frequent doses.^15,18^ Although they have a shorter half-life, *Erwinia*l-asparaginase advantage is being associated with a reduced risk of hypersensitivity reactions.^19^ The l-asparaginase enzyme from *Erwinia carotovora* subsp. *atroseptica* is an alternative to other preparations, as it has a significantly lower l-glutaminase activity than the enzymes from *E. coli* and *E. chrysanthemi.*(20,21) This is a significant factor, since l-glutaminase activity in therapeutic preparations has been associated with undesirable side effects.^1,9,11,22,23^

The production and optimization of bioproducts in large scale is a very important part of industrial production processes and is developed through knowledge of fermentation processes together with the application of recombinant DNA technology. Bioreactor cultures allow the control of important factors for microbial growth, such as temperature, pH of the media, dissolved oxygen concentration, cultivation speed and permit the addition of feed solution.^24−27^ Most fermentation processes are performed by fed-batch, due to the high productivity of biomass. The feeding strategy, as well as the feeding rate, result in the optimization of the process, as they directly affect the metabolic activity of the microorganism, leading to high yields and productivities of recombinant proteins.^24,28^ Fed-batch cultivations can be carried out using different feeding strategies, with feedback control (DO-stat or pH-stat) or without feedback control (linear or exponential pump feeding). In DO-stat cultures, feeding based on dissolved oxygen (DO) levels is used, where a feed solution is added when the DO exceeds predetermined values. In contrast, fed batches without feedback control use a feeding strategy with a linear increase in the speed of the feed pump, following the rate of cell growth.^20,24,25,29,30^

As there are few reports in the literature describing the high-yield production of l-asparaginase II, it is thus useful to develop and optimize the production of this enzyme in a recombinant form. Furthermore, to date, no work has been reported that has used these two feeding strategies (DO-stat and Linear) to obtain a high yield of the l-asparaginase II enzyme from *Erwinia carotovora* in *E. coli*. Accordingly, the objective of this work was to optimize the production of the enzyme l-asparaginase II from *E. carotovora* in *E. coli* in bioreactors using different cultivation strategies, develop a purification protocol of the recombinant enzyme and evaluate its therapeutic potential against different acute lymphoblastic leukemia cell lines.

## 2 Results and Discussion

### 2.1 Batch Cultures

**Figure 1:**
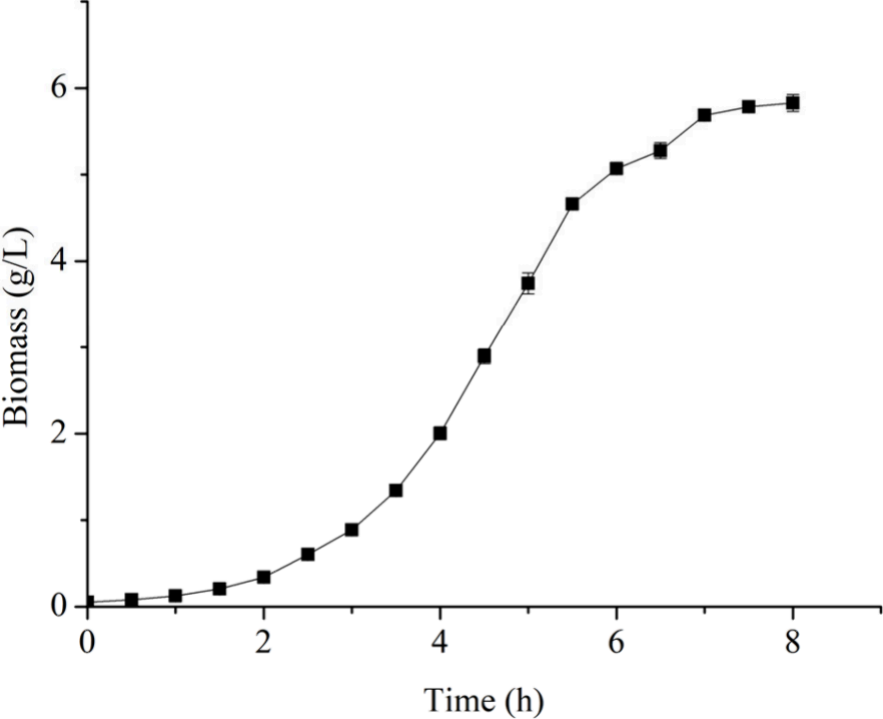
Growth curve of recombinant *E. coli* C43 (DE3) harboring a plasmid carrying a l-asparaginase gene in bioreactor batch cultivation.

Batch cultivations were carried out to establish the bacteria’s growth curve and determine the growth phases in order to define when to start feed. The growth curve of recombinant *E. coli* in a bioreactor shows that the exponential growth phase lasted up to approximately 7 h, starting from the inoculum of cells in the bioreactor (Figure 1). The analysis was carried out using a linear regression of the experimental data, with a coefficient of determination (R^2^) greater than 0.95. After this period, the cells entered the stationary phase of the growth curve, evidenced by the stabilization of cell biomass (Figure 1). The maximum biomass values were 5.83 g/L, corresponding to OD_600 nm_ 12.54 in 8 h of cultivation. Therefore, it was determined that this batch time would be the moment to start feeding the cultures in the bioreactor, in order to maximize cell growth, since the l-asparaginase product is intracellular, so usually the more cells generated the more product will be produced.

### 2.2 Fed-Batch Cultivation

**Figure 2:**
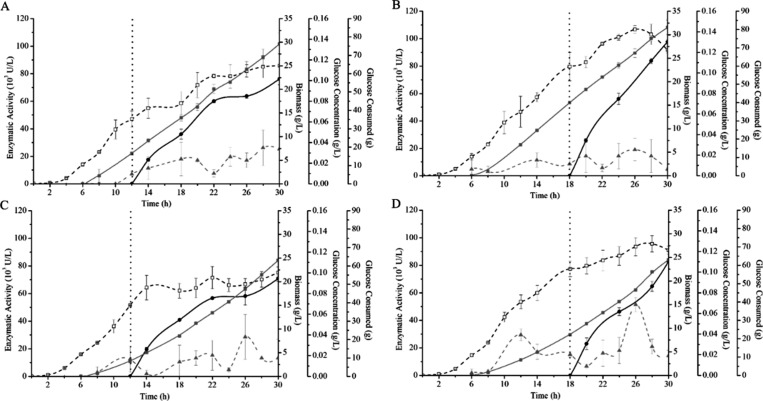
Effects of different fed-batch strategies and induction times on rASNase activity, production of biomass (g/L) and glucose consumption: (A) DO-stat, induction at 12 h of cultivation; (B) DO-stat, induction at 18 h of cultivation; (C) linear, induction at 12 h of cultivation; (D) linear, induction at 18 h of cultivation. (●) Enzyme activity (10^3^ U/L); (□) Biomass (g/L); (▲) Glucose concentration in the culture medium (g/L); (■) Consumed glucose from the start of feeding (g). Dashed line: beginning of induction with IPTG 1 mM.

The enzyme rASNase was produced in bioreactors using fed-batch cultivation. Figure 2 shows the results of enzyme activity, biomass (g/L), glucose consumption (g) and glucose concentration (g/L) in the culture medium, data obtained from the two feeding strategies used (DO-stat and linear), with induction of enzyme expression at 12 and 18 h of cultivation. In all culture conditions analyzed, the glucose in the culture medium was practically all consumed throughout the culture (30 h in total), leading to an increase in cell concentration (Figure 2). All strategies presented very low concentrations of glucose in the medium (Figure 2), with all values below 0.1 g/L, we have thus considered that glucose was completely consumed in the cultures. The DO-stat cultures showed consumption of 76.20 g for induction at 12 h (Figure 2A) and 81.15 g for induction at 18 h (Figure 2B). The linear cultures (Figure 2C and D) consumed 63 g, equally. This difference in glucose consumption is due to the DO-stat strategy as it is a direct response to cultivation, which means that when the dissolved oxygen begins to remain in the medium and exceed the set point value, the feed pump is activated and adds feed solution for the microorganisms, which in turn consume the substrate and consequently begin to increase their growth and ensuing glucose consumption. In the linear strategy, the pump is programmed with a given flow rate and the feed solution enters linearly with the speed of the feed pump throughout cultivation, independent of oxygen consumption.

Depending on the feeding strategy adopted, there may be production and accumulation of acetate in the culture medium, due to excess glucose in the medium or total consumption of dissolved oxygen, remaining close to 0% during cultivation, which can lead to inhibition of cell growth and deviation of metabolic pathways, consequently leading to a decrease in the production of recombinant proteins.^20,31^ Values larger than 0.5 g/L of acetate were observed (Table S1, Supporting Information) only in the DO-stat cultures (12 and 18 h of induction), at 8 h of growth, after which time the values did not exceed 0.28 g/L. Concentrations of 0.26 g/L or less were observed in the linear feed cultures (Table S1, Supporting Information). The presence of higher concentrations of acetate for cultures in DO-stat may have occurred due to the greater volume of feeding and concentration of glucose that entered the tank, especially at the beginning of feeding, but despite this, there was no decrease in the growth rate of the recombinant bacteria. The microbial growth of *E. coli* may be affected by the inhibitory effect of these compounds due to the reduction in pH.^32,33^ However, the low levels of acetate produced did not cause any change in the pH of the cultures, probably due to the TB culture medium, which is buffered by the phosphate solution present in its composition, and due to the automatic pH adjustment, that was carried out throughout all the cultures, thus avoiding a decrease in the cell growth rate.

In the 12 h induction cultures (Figure 2A and 2C), IPTG was added when the culture had a biomass of 13.75 g/L and 15.05 g/L (OD_600 nm_ 29.50 and 32.30), respectively. The maximum biomass values obtained in these cultures were 25.06 g/L and 21.94 g/L. In the cultures induced in 18 h (Figure 2B and 2D), the biomass at the time of induction was 23.22 g/L and 22.58 g/L (OD_600 nm_ 50.0 and 46.0), respectively. In these cultures, it was possible to observe greater cell growth, in which the maximum biomass values reached were 31.15 g/L and 27.88 g/L, with values up to 1.4-fold larger than those obtained in cultures induced in 12 h. This behavior is due to the addition of IPTG to the cultures, since the inducer can be toxic to the cells,^34,35^ resulting in a decrease in biomass concentration as was observed in the induction conditions at 12 h.

The highest rASNase yield values were found when induction was carried out at 18 h of cultivation, in both feeding strategies (DO-stat and linear) (Figure 2B and 2D), as well as total protein quantification (data not shown). The highest enzymatic activity obtained was approximately 98,000 U/L in the DO-stat controlled cultures (Figure 2B). This difference in yield observed between the strategies tested was probably due to the concentration of microbial cells, since an increase in the concentration of cells results in an increase in the production and activity of the recombinant enzyme, considering that it is expressed intracellularly.^30,36^ The condition that showed the highest biomass and enzyme activity was DO-stat with induction in 18 h (Figure 2B).

Table 1 shows the maximum values obtained for yield, productivity, growth constants and l-asparaginase production per gram of cell in fed-batch cultivations. In the cultures where induction was carried out in 18 h, in both feeding strategies, it was possible to observe a higher yield in the production of the recombinant enzyme in relation to the amount of glucose consumed (Y_P/S_) and higher biomass productivity values (Q_*x*_). Comparing the two feeding strategies with induction in 18 h, in the cultures using feedback control (DO-stat) the enzyme production yield values compared to the quantity of cells produced (Y_P/X_ = 3657.24 U/cell), as well as l-asparaginase productivity (Q_p_ = 3257.43 U/(L·h)), were approximately 1.2-fold larger than the values obtained in the linear feeding strategy (Table 1). The other parameters of specific growth rate and enzyme production were similar in all the conditions analyzed.

**Table 1:** Results of Kinetic Parameters of Fed-Batch Cultures for Production of Recombinant l-Asparaginase^a^

Feeding strategy	Induction time (h)	Y_P/S_ (U/g_glucose_)	Y_X/S_ (g_cells_/g_glucose_)	Y_P/X_ (U/g_cells_)	Q_*x*_ (g_cells_/(L·h))	Q_P_ (U/(L·h))	μ_*x*_ (h^–1^)	μ_p/x_ (h^–1^)
DO-stat	12	999.36	0.33	3046.31	0.84	2538.37	0.31	0.25
DO-stat	18	1204.23	0.33	3657.24	0.89	3257.43	0.33	0.22
Linear	12	1125.04	0.35	3236.97	0.73	2362.59	0.32	0.23
Linear	18	1313.54	0.42	3125.58	0.88	2758.43	0.32	0.22

a All parameters were calculated when the maximum rASNase activity was obtained.

Some studies have reported the production of l-asparaginase in bioreactors, using *E. coli* as the host microorganism. Roth et al.^20^ evaluated the expression of l-asparaginase II from *Erwinia carotovora* in *E. coli* C43 (DE3), using the same clone as was used in this work. The authors tested different culture media in a shaker, scaling up to a 2 L bioreactor with an exponential feeding strategy. The authors obtained a productivity of 2602.8 U/(L·h), which is 1.25 times lower than the productivity obtained in the present study. Barros et al.^36^ produced the enzyme using *E. coli* BL21 (DE3) in bioreactor, using lactose and IPTG as inducers. Maximum volumetric enzyme activity values of 43,955 U/L and biomass of 69.90 g/L were obtained when induced with lactose.^36^ Although the authors achieved a higher concentration of cells at the end of cultivation, when the yield is calculated using the ratio of maximum enzymatic activity per gram of cell produced, a value of 628.82 U/cell is obtained, almost 6-fold lower than the results obtained in this study. Mihooliya et al.^37^ expressed the l-asparaginase of *Pseudomonas resinovorans* in *E. coli* Rosetta (DE3), in a process conducted in a bioreactor with 1 L of working volume, during 24 h of experiment, with batch cultivation without feeding. A maximum enzyme activity value of 38.88 U/mL was obtained,^37^ approximately 2.5 times lower than the maximum activity obtained in this work. This difference highlights the importance of the feeding process during bioreactor cultivations, where obtaining a high cell density is related to greater productivity and yield of recombinant proteins, through the consumption of concentrated substrate in the feeding solution.^25,38,39^ The feeding condition with feedback control and induction in 18 h tested in this study proved to be satisfactory for optimizing the production of the recombinant l-asparaginase enzyme, since it was possible to obtain higher values of enzymatic activity, yields and productivities of the enzyme compared to the studies mentioned.

### 2.3 Analysis of the Cytotoxic Effect of the Purified Recombinant Enzyme in Acute Lymphoblastic Leukemia Cell Lines

**Figure 3:**
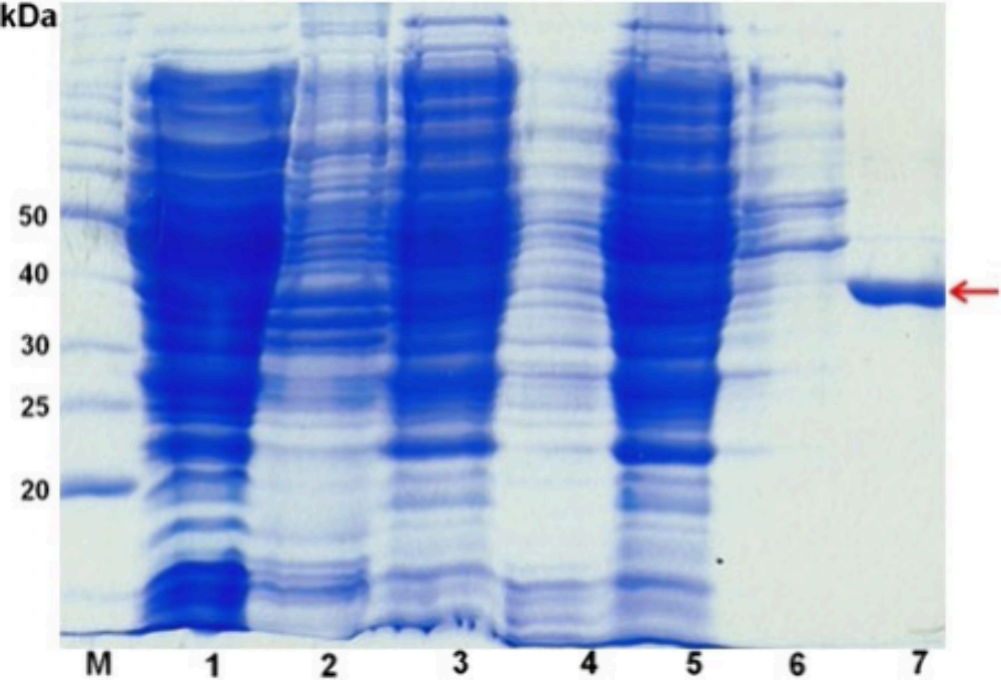
SDS-PAGE analysis of samples from the purification process steps. **M:** BenchMark Protein Ladder (molecular weight marker); Lanes: **1:** Post disruption extract, soluble fraction; **2:** Post disruption extract, insoluble fraction; **3:** Post precipitation with 1% streptomycin sulfate, soluble fraction; **4:** Post precipitation with 1% streptomycin sulfate, insoluble fraction; **5:** Extract applied to the column, post dialysis, soluble fraction; **6:** Post dialysis, insoluble fraction; **7:** Pool of eluted fractions. Corresponding enzyme band (∼36 kDa) indicated by the red arrow.

The rASNase was purified to homogeneity as described in section 4.4. The results are represented in Figure 3 and Table 2. Table 2 shows the purification protocole of the recombinant l-asparaginase enzyme. It was possible to obtain a yield of approximately 30% and 45.45-fold purification.

**Table 2:** Protein Purification Protocol of the rASNase Enzyme

Steps	Protein (mg/mL)	Total protein (mg)	Enzyme activity (U/mL)	Total activity (U)	Specific activity (U/mg)	Yield (%)	Purification fold
Crude extract	24.220	484.400	80.980	1619.60	3.34	100	
Ion-exchange column	0.211	3.165	32.030	480.45	151.80	29.66	45.45

**Figure 4:**
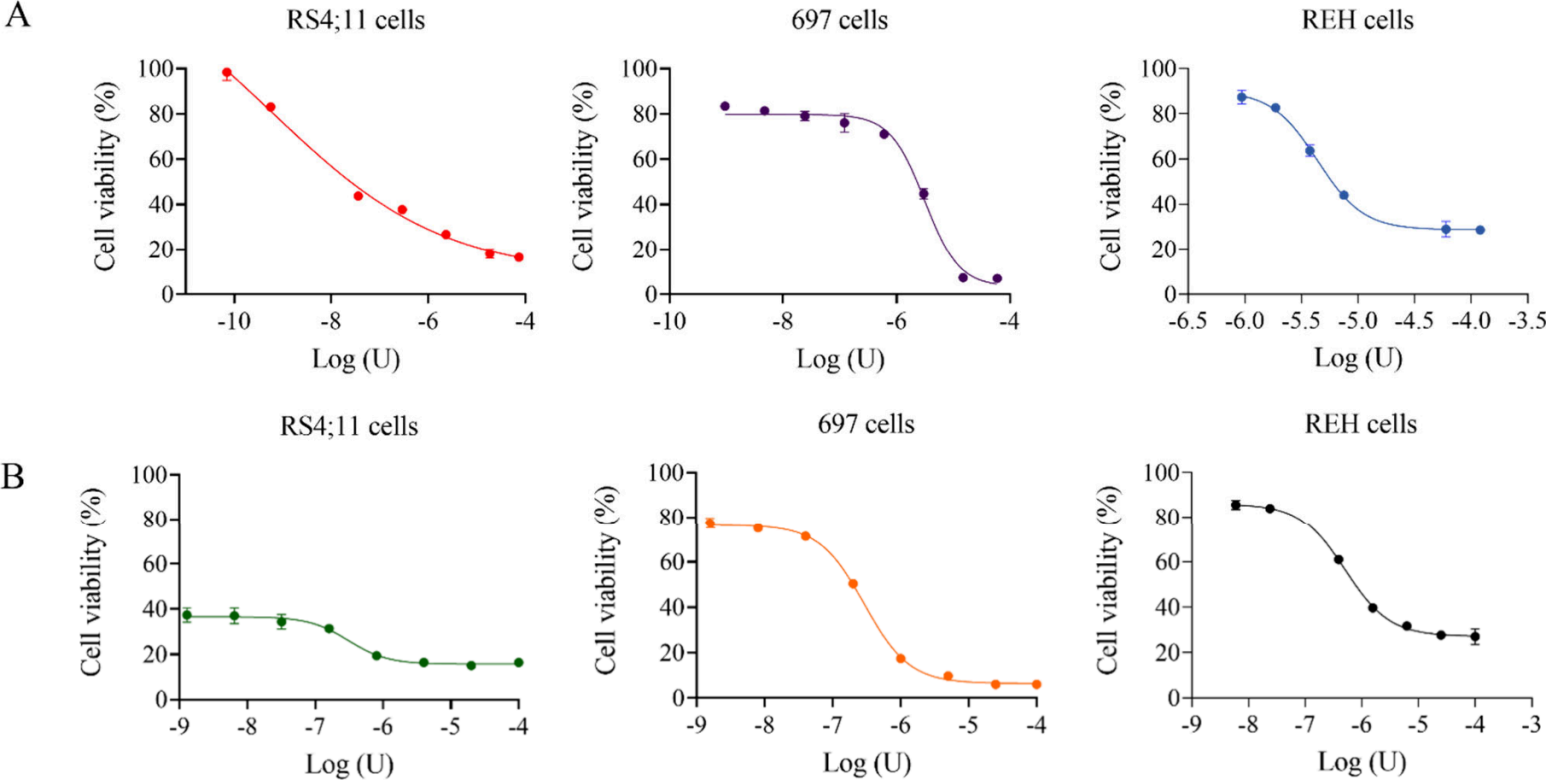
Cytotoxic effect of the recombinant enzyme (rASNase) and commercial L-ASNase (Medac) against B-cells line, measured by MTT. (A) RS4;11, 697, and REH cell viability with increased doses of rASNase. (B) RS4;11, 697, and REH cell viability with increased doses of commercial l-asparaginase (Medac).

**Figure 5:**
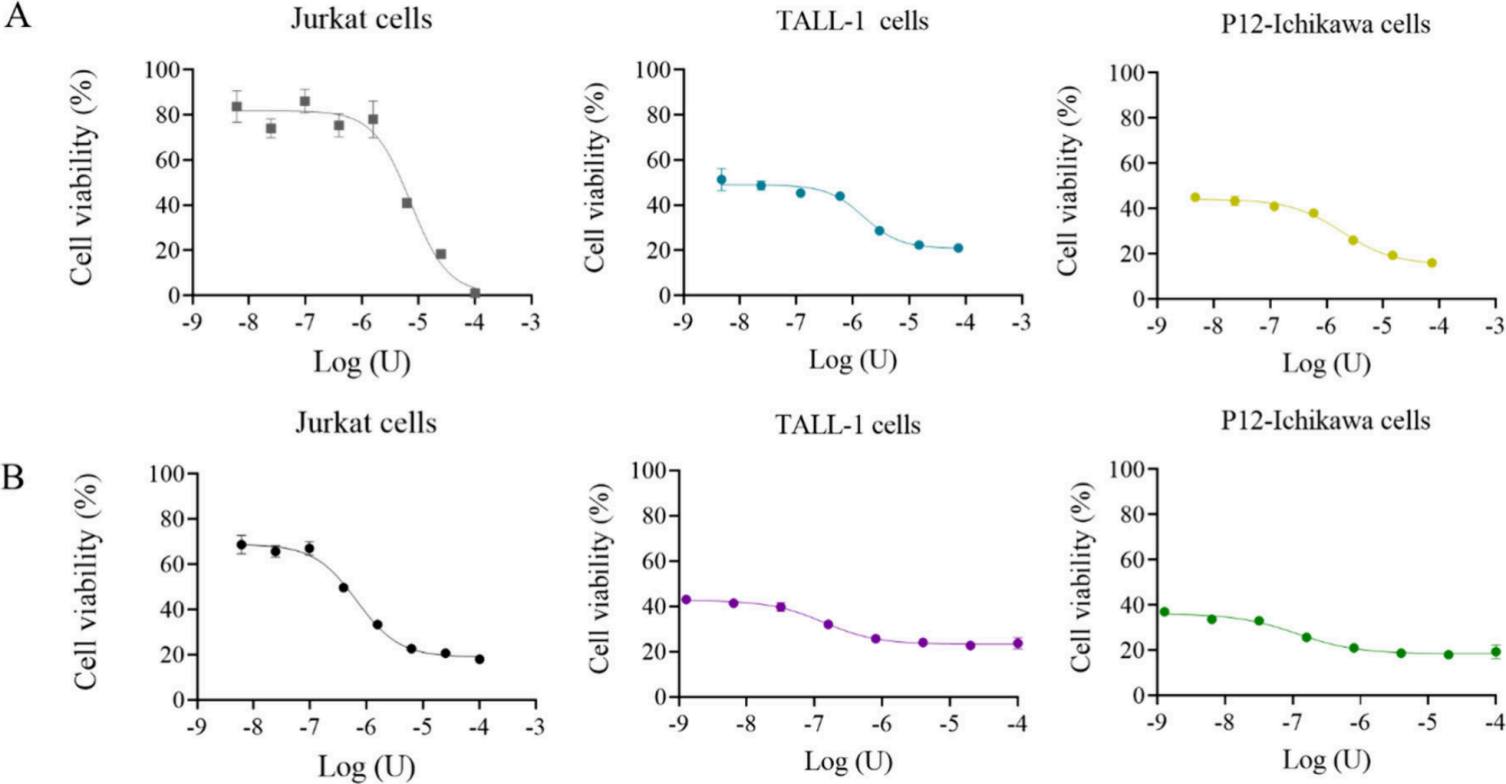
Cytotoxic effect of the recombinant enzyme and commercial l-asparaginase ASNase against T-cells line, measured by MTT. (A) Jurkat, TALL-1, and P12-Ichikawa cell viability with increased doses of rASNase. (B) Jurkat, TALL-1, and P12-Ichikawa cell viability with increased doses of commercial l-asparaginase.

**Figure 6:**
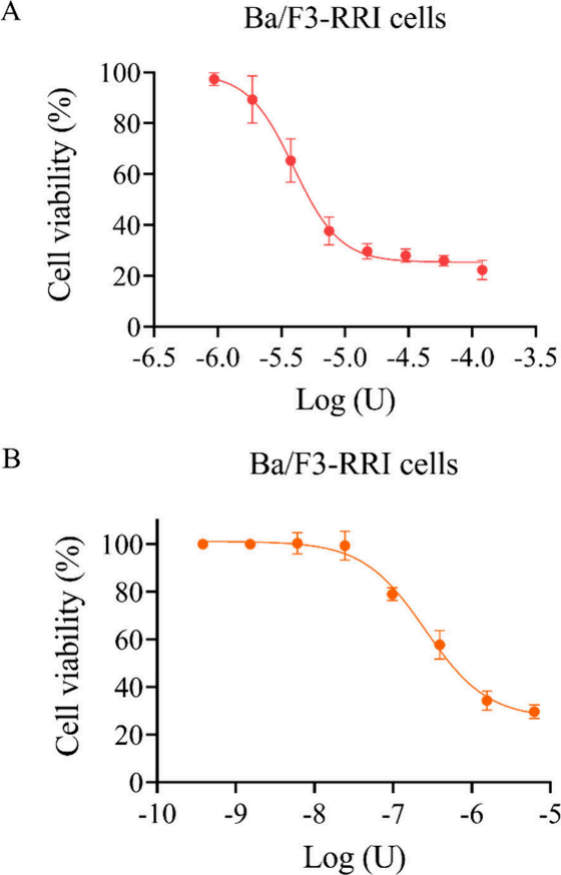
Cytotoxic effect of the recombinant enzyme and commercial l-asparaginase against leukemia/lymphoma Ba/F3 cells stably transduced with an oncogenic IL7R gene, measured by MTT. (A) Ba/F3-RRI cell viability with increased doses of rASNase. (B) Ba/F3-RRI cell viability with increased doses of commercial l-asparaginase.

The purified enzyme was used to evaluate used to evaluate its antileukemic potential. The cytotoxic effect of the recombinant enzyme was analyzed, and the results are shown in Figure 4, 5 and 6. Three human precursor B-cell lines (RS4;11, 697 and REH, Figure 4A and 4B), three human T-ALL cell lines (Jurkat, TALL-1 and P12-Ichikawa, Figure 5A and 5B) and one murine leukemia/lymphoma cell line (Ba/F3-RRI, Figure 6A and 6B) were tested. For each lineage, untreated controls, positive controls with increasing doses of the commercial enzyme (Medac) and cells treated with rASNase at increasing doses were performed, with all treatments remaining under the same experimental conditions. All obtained IC_50_ values are shown in Table 3.

**Table 3:** Values of IC_50_ Determined in the Cytotoxicity Experiment^a^

	IC_50_ (U/mL)	
Cell line	rASNase	Medac
RS4;11	5.86 × 10^–4^	<1.28 × 10^–3^
697	3.133	2.92 × 10^–1^
REH	4.381	5.04 × 10^–1^
Jurkat	7.221	6.69 × 10^–1^
TALL-1	<4.74 × 10^–3^	<1.28 × 10^–3^
P12-Ichikawa	<4.74 × 10^–3^	<1.28 × 10^–3^
Ba/F3-RRI	3.968	2.56 × 10^–1^

a The IC_50_ values were obtained using GraphPad Prism software. Medac: commercial enzyme.

The RS4;11, 697 and REH lines showed IC_50_ values for rASNase of 5.86 × 10^–4^ U/mL, 3.133 U/mL, and 4.381 U/mL, respectively (Figure 4A). Based on the results obtained, we found that the RS4;11 cell line was particularly sensitive to the purified enzyme. Parmentier et al.^3^ and Hermanova et al.^40^ described RS4;11 as being sensitive to the enzyme l-asparaginase, since these cells depend on plasma l-asparagine levels. The action of l-asparaginase enzymes reduces l-asparagine concentration leading to antitumoral activity, with inhibition of DNA and protein synthesis, causing metabolic deregulations and activation of apoptosis.^41,42^ Furthermore, this cell line can be considered asparagine synthetase (ASNS) negative, meaning that it does not express ASNS, which makes it more sensitive to the action of l-asparaginase. Extracellular l-asparagine becomes essential for the proliferation of these cell types due to the inability to synthesize this amino acid endogenously, so the enzyme’s hydrolysis mechanism leads to a reduction in extracellular l-asparagine levels, causing a decrease in the cell viability of these leukemic cells.^43,44^ The 697 and REH cell lines showed resistance to the purified enzyme (Figure 4A) as well as to the commercial enzyme (Figure 4B), showing higher IC_50_ values when compared to the values obtained with the RS4;11 cell line, for both enzymes tested. Su et al.^45^ reported resistance of the REH strain to l-asparaginase, in association with the expression of the *asparagine synthetase* gene, so that REH cells do not depend exclusively on the l-asparagine present in the extracellular microenvironment and thus l-asparagine depletion will not totally inhibit protein synthesis and consequently cell viability. Rodrigues et al.^46^ also carried out cell cytotoxicity test using the MTT method and observed that the REH cell line also was resistant to l-asparaginase enzyme, probably due to the expression of lysosomal proteases that inactivate the enzyme.

The T-ALL cell lines showed similar IC_50_ values for both the purified enzyme and the commercial enzyme (Figure 5A and B), except for the Jurkat cells which showed higher IC_50_ values (7.221 U/mL when treated with rASNase and 6.69 × 10^–1^ U/mL when treated with Medac) as found for the 697 and REH cells (Table 3). The TALL-1 and P12-Ichikawa lineages showed viabilities of less than 45% even at the lowest concentration of the enzyme used, indicating greater sensitivity. Other studies in the literature have also found greater l-asparaginase resistance by Jurkat cells.^47,48^ Abakumova et al.^47^ compared the antitumor cytotoxic response of l-asparaginase from *E. carotovora* and the enzyme derived from *E. coli* against Jurkat, finding IC_50_ values of 5 to 7.5 U/mL and 1 U/mL, respectively.

For Ba/F3-RRI, an IC_50_ value of 3.968 U/mL was obtained when rASNase was used (Figure 6A, Table 3). As can be seen in Figure 6A and B, it is possible to verify that the purified enzyme was effective in reducing the cell viability of this mouse cell line, even though it had larger IC_50_ values as seen for the 697, REH and Jurkat cell lines (Table 3).

Papageorgiou et al.^49^ observed greater cytotoxicity of the enzyme derived from *E. coli* when compared to the enzyme derived from *E. carotovora*, in agreement with the results reported in the present study. However, despite this difference in response, the authors concluded that the *Erwinia* enzyme had a satisfactory inhibitory effect on the growth of leukemia cells (Raji and MOLT-4 cells), observing a significant decrease in cell viability,^49^ similar to that found in our studies. The difference in the efficiency of *Erwinia*l-asparaginase compared to *E. coli*-derived enzyme was expected, since the half-life of the former is shorter than the latter, in addition to other factors that still need further elucidation, such as glutaminase activity.^9,42,50,51^ In the study by Grima-Reyes et al.,^52^ the authors suggest that the l-glutaminase activity of ASNase prevents possible resistance, resulting in greater efficiency of the enzyme in the treatment of leukemia. A combination of asparaginase and glutaminase activity could provide greater antitumor effects by targeting two amino acids, asparagine and glutamine. Other authors have also reported that the glutaminase activity of ASNase may be necessary for efficient anticancer activity in cells expressing low or no levels of ASNS.^44,51^ In the case of the *E. coli*-derived enzymes, it is already known that they have higher glutaminase activity when compared to the *E. carotovora*-derived enzyme,^20^ so this could also be a factor that explains the greater efficiency of the commercial enzyme (derived from *E. coli*) in the present cytotoxicity experiments. The *E. coli* enzyme is used as a first-line drug because of its high efficiency, but when there are hypersensitivity reactions and no response to treatment, there is a need to change the treatment approach and use other enzymes, with *Erwinia*l-asparaginase being a suitable replacement. Some approaches can be adopted to improve the anticancer activity of the l-asparaginase enzyme derived from *E. carotovora*. These approaches include increasing thermal stability and half-life, factors that influence the enzyme’s antileukemic potential.^53,54^

In the present study, all the cell lines tested showed a decrease in cell viability and the toxicity was found to be dose-dependent, since an increase in growth inhibition was observed as the concentration of the drugs tested increased. IC_50_ calculations were carried out based on the nonlinear nature regression of drug dose–response studies.

**Figure 7:**
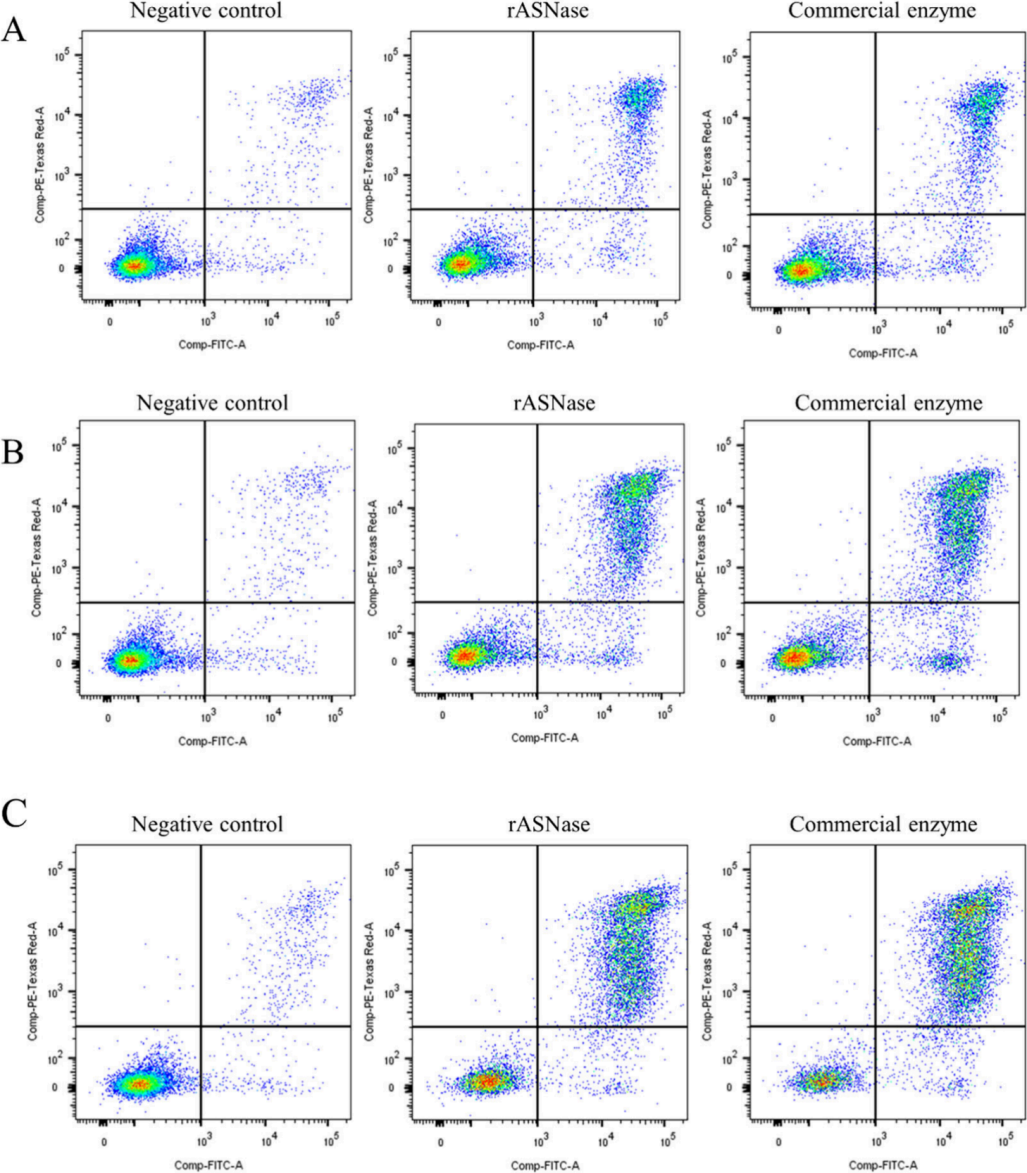
Flow cytometry analysis of cells incubated with the IC_50_ dose of rASNase and commercial enzyme and labeled with Annexin V/PI. (A) RS4;11 cells after 24 h treatment with l-asparaginases. (B) RS4;11 cells after 48 h treatment with l-asparaginases (middle panels). (C) RS4;11 cells after 72 h treatment with l-asparaginases (right panels). Negative controls are the cells without treatment (left panels). Living cells (lower left quadrants), early apoptotic cells (lower right quadrants), late apoptotic cells (upper right quadrants) and death cells (upper left quadrants) are presented.

Since RS4;11 cell line showed greater sensitivity to the rASNase, we used these cells in the cell death test. The cells were incubated at the IC_50_ concentrations previously determined in the cytotoxicity experiments and were analyzed at 24 h, 48 and 72 h, as shown in Figure 7A, B and C, together with untreated negative controls (left panels). Cell death by apoptosis (A+/P- or A+/PI+) could be seen at all the times analyzed. The dual-labeling assay can accurately detect and quantify the percentage of live cells, early apoptotic cells, and late apoptotic cells. The results demonstrated that the recombinant l-asparaginase from *E. carotovora* induced apoptosis of RS4;11 leukemia cells (Figure 7).

**Figure 8:**
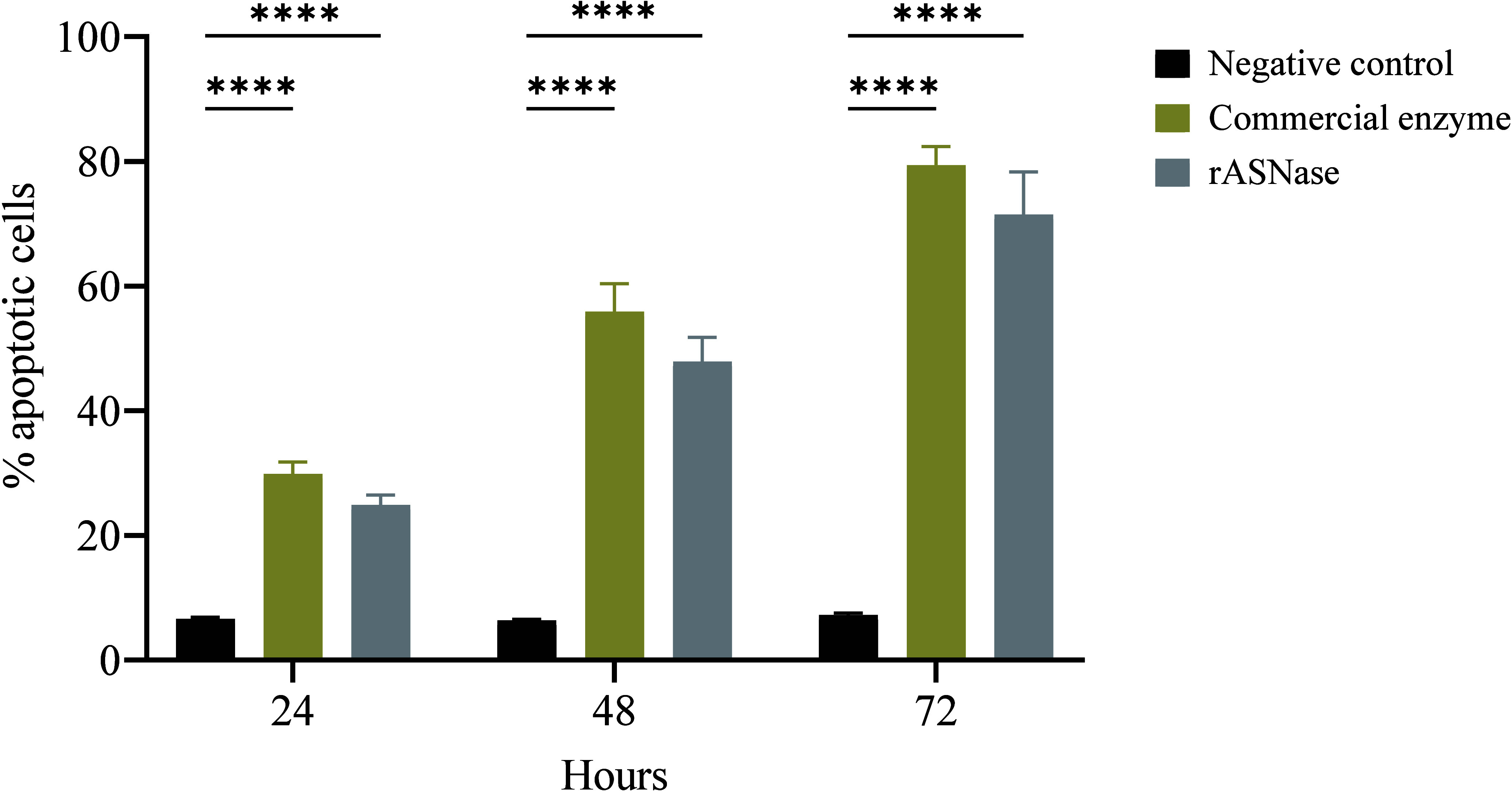
Apoptotic effect of recombinant and commercial l-asparaginase on RS4;11 cell line. Cells were plated in triplicate and treated with IC_50_ values of rASNase and commercial enzyme. Negative control are the cells without treatment. The values shown are the mean ± standard deviation of triplicate experiment. Statistical analysis was determined by two-way analysis of variance (ANOVA), followed by Tukey’s multiple comparisons test using GraphPad Prism software. ****p < 0.0001.

The staining with Annexin V/PI showed a percentage of apoptotic cells of approximately 25%, 48% and 71.5% after, respectively, 24 h, 48 and 72 h of treatment with rASNase (Figure 8). The use of the commercial enzyme (Medac) as a positive control resulted in a percentage of apoptotic cells similar to that found with the *E. carotovora* enzyme, with approximately 30%, 55% and 79.4% after, respectively, 24 h, 48 and 72 h of treatment (Figure 8). In this context, rASNase proved to be a potent therapeutic agent against leukemic cells. Consistent with the enzyme’s proposed mechanism of action, cells were killed by apoptosis. Depletion of l-asparagine by the recombinant enzyme and the inability of the leukemia cells to synthesize their own l-asparagine results in the interruption of protein synthesis that led to apoptosis induction.^55^ The results demonstrate that the recombinant enzyme exhibits cytotoxic activity and induces apoptosis in leukemia cells.

## 3 Conclusion

In this work, different bioreactor cultivation strategies were studied to optimize the production of the recombinant enzyme l-asparaginase II from *Erwinia carotovora* in *Escherichia coli*. The use of the DO-stat and linear feeding control strategies to produce the *E. carotovora*l-asparaginase II was reported for the first time. In previous studies found in the literature, production has been carried out in a bioreactor, but other feeding strategies have been tested, which can lead to some limitations in terms of yield and productivity. Both strategies used in this study were efficient in obtaining higher yields, without leading to the accumulation of metabolites that could inhibit cell growth. The DO-stat strategy with induction in 18 h was defined as the best strategy for increasing l-asparaginase production, observing a productivity of 3257.43 U/Lh, with a maximum activity of approximately 98,000 U/mL. The recombinant enzyme produced was used in *in vitro* tests to verify its antileukemic potential against different types of ALL. The enzyme proved to be efficient in reducing the cell viability of leukemia cells, as well as inducing apoptosis. These results offer a solid foundation for the development of much needed alternatives for the treatment of ALL.

## 4 Materials and Methods

### 4.1 Materials

The recombinant protein expression experiments were carried out using *E. coli* C43 (DE3) host cells bearing the *Erwinia carotovora*l-asparaginase II coding gene cloned into pET30a(+) expression vector (Novagen). This construction was previously cloned and reported by Roth et al.^20^ Kanamycin, Isopropyl β-D-thiogalactoside (IPTG), l-asparagine were obtained from Sigma-Aldrich (Missouri, US), and the culture media from Merck (Darmstadt, Germany) and Invitrogen (Thermo Fisher Scientific, Massachusetts, US). The other substances used were analytical-grade reagents (Sigma-Aldrich, Missouri, US)

### 4.2 Methods

#### 4.2.1 Bioreactor Experiments

The cultivation conditions such as culture medium, growth temperature, IPTG inducer concentration and the expression strain for *E. carotovora*l-asparaginase II enzyme (rASNase) were determined by Roth et al.^20^ These conditions were used to produce the enzyme in the present study.

The production of rASNase was carried out in a Biostat B Plus Bioreactor (Sartorius Stedim, Germany), in batch and fed-batch cultivations, with two 2-L stirred tank, filled with 1 L of culture medium. The bioreactor was equipped with two six-flat-blade turbines, and with stirrer speed, air flow rate, temperature and pH electrodes (EasyFerm Plus k8 200, Hamilton Company). A polarographic electrode was used to measure the dissolved oxygen concentration (OxyFerm FDA 225, Hamilton Company) in the culture. Cultivations were carried out at 30 °C, stirring at 300 to 1000 rpm, pH 7.0 automatically controlled using 10% (v/v) orthophosphoric acid and 12.5% (v/v) ammonium hydroxide and manual addition of antifoam was done when necessary. The online monitoring system recorded measurements for pO_2_, pH, stirrer speed, base and acid consumption, and aeration rate through an external data acquisition and control system (Sartorius Stedim, Germany).

A master cell bank (MCB) containing the selected *E. coli* C43 (DE3) strain was prepared in 40% glycerol and stored at −20 °C and −80 °C. The MCB was grown on Petri plates to obtain isolated colonies of the bacteria. The bioreactor preinoculum 1 was prepared by inoculating an isolated colony in 20 mL of LB medium (10 g/L NaCl, 10 g/L tryptone, 5 g/L yeast extract), containing 30 μg/mL kanamycin. The culture was grown overnight in a shaker at 180 rpm at 37 °C. This culture was then used to prepare the preinoculum 2 at an optical density at 600 nm (OD_600 nm_) of 0.1, in a volume of 100 mL of LB medium containing appropriate antibiotic. The cells were incubated on an orbital shaker (180 rpm) at 37 °C until they reached an optical density of 1.0. The bioreactor was inoculated with the preinoculum 2 with final OD_600 nm_ of 0.1.

##### 4.2.1.1 Batch Cultivation

The batch cultures were carried out with 900 mL of TB medium (12 g/L tryptone, 24 g/L yeast extract, 12.5 g/L K_2_HPO_4_, 2.3 g/L KH_2_PO_4_, 4 mL/L glycerol), 30 μg/mL kanamycin and 100 mL of preinoculum 2 at OD_600 nm_ 1.0, as described in section 4.2.1. Cultivations were conducted at 30 °C, keeping dissolved oxygen at 30% varying stirrer speed (300–1000 rpm) with constant air flow rate of 1 vvm (air volume per volume of culture medium per minute). Samples were collected every 30 min, and the cell concentration (biomass) of appropriately diluted culture samples was measured by optical density at 600 nm using a spectrophotometer, previously zero-calibrated with culture supernatant. The measurements were performed with disposable plastic cuvettes with a path length of 1 cm. Cultivations were terminated when the cells reached the stationary phase, indicated by constant biomass. This step was carried out to determine when to start feeding the fed-batch cultures. The batch cultures were made in triplicate.

##### 4.2.1.2 Fed-Batch Cultivation

The fed-batch cultivations were based on some parameters reported by De Andrade et al.^24^ and Roth et al.^20^ The cultures were started with 900 mL of TB medium, 30 μg/mL kanamycin and 100 mL of preinoculum 2 (1 L working volume), prepared according to the methodology described in section 4.2.1. Dissolved oxygen was maintained at 30% throughout the cultivation, with a constant air flow rate of 1 vvm. Feeding was started at 7 h of cultivation, at the end of the exponential growth phase, and the total time of the experiment was 30 h. The feeding solution used consisted of TB 2x concentrate, 300 g/L glucose, 40 mM magnesium sulfate and 30 μg/mL kanamycin. Two feeding strategies were adopted: (I) dissolved oxygen feedback control (DO-stat) maintained at 30%, with a gradual increase of stirring from 300 rpm to constant speed of 1000 rpm; (II) without feedback control with a linear feed flow rate of 1 to 3% of the total pump flow (7.6 mL/min), with dissolved oxygen maintained at 30% by cascade stirring (300 to 1000 rpm). Different times of induction with IPTG (12 and 18 h after the initiation of cultivation) were tested to evaluate the effect of induction time on the expression of recombinant l-asparaginase. During cultivation, samples were collected at determined times to evaluate cell growth, obtain and determine biomass (dry cell weight), assess recombinant enzyme production, and analyze metabolites, and stored at −20 °C. All experiments were carried out in duplicate.

#### 4.2.2 Analytical Methods

##### 4.2.2.1 Quantification of Cell Concentration

Samples were collected throughout cultivation and the optical density of growth was measured at 600 nm. To determine the dry cell mass, aliquots of known volume were centrifuged (6000 rpm, 10 min, room temperature) in previously weighed and labeled tubes. The pellet was resuspended in 50 mM phosphate buffer (pH 7.2), centrifuged again and the resulting pellet was dried at 80 °C until constant mass. The tubes were then reweighed, the difference obtained divided by the aliquoted volume and defined as the total concentration of cells corresponding to the optical density measured for each sample. One optical density unit was found to be equivalent to 0.465 g/L of dry cell mass per volume.

##### 4.2.2.2 Determination of Enzymatic Activity and Quantification of Total Proteins

Samples were periodically collected to determine enzyme activity and quantify total proteins. The aliquots were standardized to OD_600 nm_ 20 and centrifuged (6000 rpm, 10 min, room temperature). Recombinant cells were resuspended in 50 mM potassium phosphate pH 7.5, disrupted by ultrasonication (Sonics Vibra-Cell VCX 750, Sonics & Materials, Connecticut, US) at a range of 60%, with eight 10 s pulses, with 1 min ice bath between each pulse. The soluble fraction was separated by centrifugation (at 4 °C, 38900 × *g*, for 30 min) and analyzed. The enzymatic activity of rASNase (U/L) was assessed using l-asparagine as a substrate. Quantification was carried out by detecting the formation of ammonia resulting from the hydrolysis of the substrate, using Nessler’s reagent, according to the methodology of Imada et al.,^56^ Shifrin et al.^57^ and Zhang et al.,^58^ with modifications. A calibration curve was constructed from a 6 mM solution of ammonium sulfate ((NH_4_)_2_SO_4_), diluted to concentrations ranging from 0 to 0.208 μmol of ammonium ion (NH_4_^+^). The curve consisted of the reaction of 40 μL of the solution at the different concentrations, 860 μL of 50 mM potassium phosphate pH 7.5 and 100 μL of Nessler’s reagent. The absorbance of curve samples was measured in a spectrophotometer at 436 nm.

To analyze the samples obtained from the cultures, the soluble fraction obtained after cell rupture was used to conduct two reactions: (1) 588 μL of 50 mM potassium phosphate buffer pH 7.5, 400 μL of l-asparagine (10 mM) and 12 μL of sample were added to a microtube. The reaction was incubated at room temperature for 7 min and stopped by adding 100 μL of 20% Trichloroacetic acid (TCA); (2) the second reaction consisted of adding 860 μL of 50 mM potassium phosphate buffer pH 7.5, 40 μL of reaction 1 and 100 μL of Nessler’s reagent to a microtube and mixing by inversion. The colorimetric reaction was measured in a spectrophotometer at 436 nm. One unit of activity (U) was defined as the amount of enzyme that catalyzes the formation of 1 μmol of ammonia from l-asparagine per minute under the assay conditions.

The total protein concentration was analyzed by Bradford reagent (Quatro G Biotecnologia, RS, Brasil), using bovine serum albumin as standard,^59^ at concentrations of 0.1 mg/mL to 1.0 mg/mL. Readings were measured on a plate reader (EZ Read 400, Biochrom, Cambridge, UK) at 595 nm.

##### 4.2.2.3 Acetic Acid Analysis by Gas Chromatography Coupled to Mass Spectrometry (GC/MS) and Glucose Quantification by Enzymatic Method

Supernatants obtained after centrifugation of cultivation cell samples were analyzed for glucose and acetate concentration. The chromatographic analyses were carried out using gas chromatography (Agilent, GC8890 GC System, Santa Clara, USA) coupled to mass spectrometry (Agilent, 5977B GC/MSD, Santa Clara, USA), equipped with a quadrupole analyzer and an electrical ionization source. The samples were injected with an autosampler (Agilent, PAL RSI 85, Santa Clara, USA) onto a DB-FATWAX UI column (30 m x 0.25 mm × 0.25 μm) (Agilent, Santa Clara, USA). The transfer line, ionization source and quadrupole temperatures were maintained at 290, 280, and 150 °C, respectively. Helium was used as the carrier gas at 1.5 mL/min.

Sample preparation for acetate quantification was performed by 1 mL of the supernatant obtained after centrifugation of the microbial cells. The supernatant was recentrifuged and transferred to a microtube containing 6 mg of oxalic acid. To determine acetate, the column temperature was held at 90 °C for 6 s, heated to 120 °C at 60 °C/min and held for 1 min, with subsequent heating to 140 °C at 30 °C/min, maintained for 1 min, followed by a gradual increase to 250 °C at 40 °C/min and then maintained for 7 min. The samples (0.5 μL) were injected in splitless mode, with an inlet temperature of 280 °C. The MS was maintained in Scan mode from 15 to 300 *m*/*z* and ionization energy of 70 eV. Acetate was quantified using calibration curves (20 to 750 mg/L), using MassHunter Quantitative Analysis 10.0 software (Agilent, Santa Clara, US). The analyses were carried out in triplicate.

The glucose concentration in the bioreactor cultivation was assessed using a colorimetric enzyme kit based on oxidase-peroxidase (Labtest, Glucose Liquiform, Labtest, Lagoa Santa, Brazil), according to the manufacturer’s manual.

### 4.3 Kinetic Parameters of the l-Asparaginase Production Process in the Bioreactor

The equations bellow were used to calculate the following cultivation parameters: l-asparaginase yield per substrate (Y_P/S_) (eq 1), biomass yield per substrate (Y_X/S_) (eq 2), l-asparaginase yield per biomass (Y_P/X_) (eq 3), biomass productivity (Q_X_) (eq 4), l-asparaginase productivity (Q_P_) (eq 5), specific cell-growth rate (μ_X_) (eq 6), and specific l-asparaginase production rate per biomass (μ_P/X_) (eq 7).^60,61^ All kinetic parameters were calculated when the maximum rASNase activity was obtained:
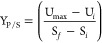
1
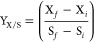
2
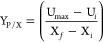
3
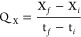
4
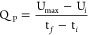
5
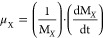
6
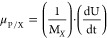
7where U_*max*_ = maximum l-asparaginase activity (U/L), U_*i*_ = initial l-asparaginase activity (U/L), S_*f*_ = glucose concentration (g/L) when the maximum activity was obtained, S_*i*_ = initial glucose concentration (g/L), X_*i*_ = initial biomass concentration (g/L), X_*f*_ = biomass concentration (g/L) when the maximum activity was obtained, t_*f*_ = cultivation time (h) when the maximum activity was obtained, t_*i*_ = initial cultivation time (h), and M_X_ = cell mass (g).

### 4.4 Purification of rASNase by High Performance Liquid Chromatography

The recombinant l-asparaginase enzyme was purified by high-performance liquid chromatography (HPLC), using an ion exchange purification protocol run in an AKTA system (GE Healthcare, UK), at 4 °C. *E. coli* cells stored at −20 °C were resuspended in 20 mM potassium phosphate buffer pH 5.5 (buffer A) (1g wet cell weight/10 mL of buffer A) and disrupted in a French press (Constant Cell Disruption Systems, UK), with local pressure of 30 kpsi (206.8 bar). The cell lysate was centrifuged (4 °C, 38900 × *g*, for 30 min) and the supernatant was incubated with 1% (v/v) streptomycin sulfate solution under gentle stirring at 4 °C for 30 min. The solution was centrifuged again (4 °C, 38900 × *g*, for 30 min) and the resulting supernatant was dialyzed twice against 2 L of buffer A. The dialyzed cell extracts were then centrifuged (4 °C, 38900 × *g*, for 30 min) and the supernatants were loaded on a Resource S cation exchange chromatography column (Cytiva Life Sciences, Malborough, US) equilibrated with buffer A. The sample was applied at a flow rate of 2 mL/min, the unbound proteins were washed with 5 column volumes (CV) at a flow rate of 6 mL/min and the adsorbed proteins were eluted with a linear pH gradient with 20 CV, from pH 5.5 to 8.5 of 20 mM potassium phosphate buffer. The eluted fractions containing the recombinant enzyme were pooled, analyzed for enzymatic activity and protein concentration, then frozen at −20 °C and lyophilized (ModulyoD, Thermo Scientific, US) at 4 mbar at −35 °C for 24 h. The purification result was visualized by polyacrylamide gel electrophoresis (SDS-PAGE 12%) and stained with Fast Stain Comassie SDS-PAGE (Quatro G Biotecnologia, RS, Brazil).

### 4.5 Analysis of the Cytotoxic Effect of the Recombinant Enzyme on Acute Lymphoblastic Leukemia Cell Lines

#### 4.5.1 Cytotoxicity Assay Using the MTT Method

To determine the therapeutic potential of purified rASNase, the antileukemic effect was evaluated against different acute lymphoblastic leukemia (ALL) cell lines. The ALL-cell lines were grown in RPMI-1640 culture medium supplemented with 10% fetal bovine serum, 100 UI/mL penicillin, 100 pg/mL streptomycin and maintained at 37 °C and 5% CO_2_. The experiments were conducted with 3 human precursor B-cell ALL strains (REH, RS4;11 and 697), 3 human T-cell ALL strains (Jurkat, TALL-1 and P12-Ichikawa) and 1 mouse leukemia/lymphoma strain transduced with the IL7R oncogenic receptor (Ba/F3-RRI;^62^). Cytotoxicity assays were performed using the MTT method. The cells were resuspended at a concentration of 30,000 cells in 80 μL of culture medium and seeded in 96-well plates. The lyophilized enzyme was reconstituted with phosphate-buffered saline (PBS), then concentrated using Amicon Ultra Centrifugal Filter 10 kDa MWCO (Merck, Germany) and the enzyme activity was measured using the substrate l-aspartic beta-hydroxamate (AHA), following the methodology proposed by Lanvers et al.,^63^ with modifications. The standard curve for calculating enzyme activity was prepared using the commercial l-asparaginase from *E. coli* (Medac GmbH, Germany). Increasing doses of rASNase and commercial l-asparaginase Medac (positive control), in 20 μL, were added per well, in triplicate. After 48 h incubation at 37 °C and 5% CO_2_, 10 μL of tetrazolium salt solution (MTT) (5 mg/mL in PBS) was added, followed by further incubation for 4 h at 37 °C and 5% CO_2_. Afterward, 100 μL of acid SDS (10% sodium dodecyl sulfate, 0.1 M HCl) was added to dissolve the formazan crystals. After overnight incubation, absorbance was read at 570 nm using the Synergy H1 Hybrid Reader (BioTek). The percentage of viable cells was calculated according to eq 8. The IC_50_ values were obtained using GraphPad Prism software.
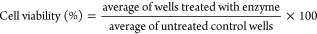
8

#### 4.5.2 Evaluation of Cell Death by Annexin V/Propidium Iodide Assay

The RS4;11 cell line was used for the cell death assay. The cells were grown at 150,000 cells per well in 48-well plates in RPMI-1640 medium plus 10% fetal bovine serum, 100 IU/mL penicillin, 100 pg/mL streptomycin. In the wells, 40 μL of the enzymes rASNase or commercial l-asparaginase Medac were added at the IC_50_ doses determined in the cell viability assay. Time points of 24 h, 48 and 72 h and nondrug controls were conducted under the same assay conditions. After the treatment times, the cells were washed with 2 mL of PBS, centrifuged at 400 × *g* for 5 min and resuspended in 100 μL of 1x Annexin V Binding Buffer. The cells were incubated with 3 μL of FITC Annexin V (BD Biosciences, US) and remained at room temperature for 15 min, protected from light. Then 250 μL of Annexin V Binding buffer and 5 μL of propidium iodide (PI) (300 μg/mL) were added and incubated at room temperature for 3 min. The samples were analyzed on the LSRFortessa Cell Analyzer (BD Biosciences, US) using FlowJo Software (BD Biosciences). All experiments were performed in triplicate.

## Supporting Information Available

Table (S1) of acetate concentrations produced in the different culture conditions of recombinant *Escherichia coli* (PDF)
