# Supplementary material for: Production Process Optimization of Recombinant Erwinia carotovoral-Asparaginase II in Escherichia coli Fed-Batch Cultures and Analysis of Antileukemic Potential
Source: ACS Omega. 2024 Aug 3;9(32):34951–63. doi: 10.1021/acsomega.4c04711 (PMC11325515; doi:10.1021/acsomega.4c04711)
Supplement: Supplementary file 1 — ao4c04711_si_001.pdf [file ao4c04711_si_001.pdf]

Supporting Information

Production process optimization of recombinant *Erwinia carotovora* L-asparaginase II in *Escherichia coli* fed-batch cultures and analysis of antileukemic potential

Bruna Coelho de Andrade[a,b], Gaby Renard[c], Adriano Gennari[d], Leonardo Luís Artico[e,f], José Ricardo Teixeira Júnior[e,f], Daniel Kuhn[d], Priscila Pini Zenatti Salles[e,f], Claucia Fernada Volken de Souza[d], Gustavo Roth[g], Jocelei Maria Chies[c], José Andrés Yunes[e,h**], Luiz Augusto Basso[a,b,i*]

[a] National Institute of Science and Technology in Tuberculosis, Research Center for Molecular and Functional Biology, Pontifical Catholic University of Rio Grande do Sul, Porto Alegre, RS, Brazil.

[b] Graduate Program in Medicine and Health Sciences, Pontifical Catholic University of Rio Grande do Sul, Porto Alegre, RS, Brazil.

[c] Quatro G Pesquisa & Desenvolvimento Ltd., Porto Alegre, RS, Brazil.

[d] Food Biotechnology Laboratory, Biotechnology Graduate Program, University of Vale do Taquari (UNIVATES), Lajeado, RS, Brazil.

[e] Centro Infantil Boldrini, Campinas, SP, Brazil

[f] Graduate Program in Genetics and Molecular Biology, Biology Institute, State University of Campinas, Campinas, SP, Brazil

[g] Pontifical Catholic University of Rio Grande do Sul, Porto Alegre, RS, Brazil.

[h] Department of Medical Genetics, Faculty of Medical Sciences, State University of Campinas, Campinas, SP, Brazil.

[i] Graduate Program in Cellular and Molecular Biology, Pontifical Catholic University of Rio Grande do Sul, Porto Alegre, RS, Brazil

* Corresponding author. National Institute of Science and Technology in Tuberculosis, Research Center for Molecular and Functional Biology, Pontifical Catholic University of Rio Grande do Sul, Porto Alegre, RS, 90616-900, Brazil. E-mail address: luiz.basso@pucrs.br

** Corresponding author. Centro Infantil Boldrini, 1270 Dr. Gabriel Porto St, Campinas, SP, 13083-210, Brazil. E-mail address: andres@boldrini.org.br

Table S1. Acetate concentrations in different culture conditions of recombinant *Escherichia coli.*

Feeding strategy	Induction Time (h)	Time of cultivation (h)	Acetate (g/L)
DO-stat	12	8	0.608 ± 0.02
		12	0.153 ± 0.01
		14	0.140 ± 0.00
		18	0.263 ± 0.15
		20	0.277 ± 0.15
		22	0.264 ± 0.00
		24	0.205 ± 0.04
		26	0.192 ± 0.01
		28	0.237 ± 0.03
		30	0.225 ± 0.01
DO-stat	18	8	0.528 ± 0.17
		12	0.204 ± 0.03
		14	0.207 ± 0.02
		18	0.222 ± 0.03
		20	0.209 ± 0.04
		22	0.231 ± 0.01
		24	0.258 ± 0.01
		26	0.236 ± 0.02
		28	0.250 ± 0.03
		30	0.236 ± 0.05
Linear	12	8	0.267 ± 0.04
		12	0.116 ± 0.03
		14	0.148 ± 0.00
		18	0.217 ± 0.12
		20	0.163 ± 0.05
		22	0.151 ± 0.02
		24	0.160 ± 0.02
		26	0.165 ± 0.01
		28	0.180 ± 0.01
		30	0.208 ± 0.01
Linear	18	8	0.230 ± 0.02
		12	0.135 ± 0.05
		14	0.125 ± 0.02
		18	0.152 ± 0.01
		20	0.143 ± 0.02
		22	0.159 ± 0.03
		24	0.166 ± 0.00
		26	0.256 ± 0.08
		28	0.245 ± 0.04
		30	0.266 ± 0.08

Results expressed as mean ± standard deviation.
